# Targeting Oral Pathogens with *Salvia officinalis* and *Nigella sativa* Supercritical CO_2_ Extracts: A Pharmacodynamic Approach and Three-Dimensional Checkerboard Synergy for Novel Dental Antimicrobials

**DOI:** 10.3390/antibiotics14111100

**Published:** 2025-11-02

**Authors:** Luka Tucakov, Ana Tomić, Olja Šovljanski, Milica Aćimović, Ana Miljković

**Affiliations:** 1 Department of Dentistry, Faculty of Medicine, University of Novi Sad, 21000 Novi Sad, Serbia; drlukatucakov@gmail.com; 2 Department of Maxillofacial Surgery, University Clinical Centre of Vojvodina, 21000 Novi Sad, Serbia; 3 Faculty of Technology Novi Sad, University of Novi Sad, 21000 Novi Sad, Serbia; oljasovljanski@uns.ac.rs; 4 Institute of Field and Vegetable Crops Novi Sad, Maksima Gorkog 30, 21000 Novi Sad, Serbia; milica.acimovic@ifvcns.ns.ac.rs; 5 Department of General Medicine and Geriatrics, Faculty of Medicine, University of Novi Sad, 21000 Novi Sad, Serbia; ana.miljkovic@mf.uns.ac.rs

**Keywords:** sage, black cumin, CO_2_ extract, antimicrobial activity, oral biofilm, *Streptococcus mutans*, natural dental therapeutics

## Abstract

**Background**: Oral infections such as dental caries and candidiasis are mediated by resilient biofilms, which are increasingly tolerant to conventional antimicrobials. This study investigated the antimicrobial and antibiofilm effects of *Salvia officinalis* and *Nigella sativa* CO_2_ extracts against *Streptococcus mutans* and *Candida albicans*, with emphasis on synergistic interactions. **Methods**: Extracts were analyzed using gas chromatography–mass spectrometry analysis (GC–MS) and evaluated through planktonic MIC/MBC assays, time–kill kinetics, and biofilm models (MBIC/MBEC, biomass, metabolic activity). A novel three-dimensional checkerboard (3D-CB) and fractional inhibitory concentration index (FICI) approach was applied to optimize extract ratios, concentrations, and exposure times. **Results**: *S. officinalis* extract showed greater activity against *S. mutans* (MIC 256 mg/L; MBC 512 mg/L), while *N. sativa* was more effective against *C. albicans* (MIC 256 mg/L; MFC 512 mg/L). Both extracts reduced biofilm biomass and metabolic activity by over 70% at higher doses. Synergy was confirmed at ratios of 70:30 (*S. officinalis: N. sativa*) for *S. mutans* (FICI 0.38) and 40:60 for *C. albicans* (FICI 0.42). The achieved synergistic effect further decreased MBEC values fourfold and prolonged post-antibiotic effects. **Conclusions**: Synergistic *S. officinalis*–*N. sativa* formulations enhanced antimicrobial activity against oral pathogens in both planktonic and biofilm states, supporting their potential as next-generation dental antimicrobials.

## 1. Introduction

Oral diseases such as caries, gingivitis, and periodontitis remain persistent global challenges, driven by the activity of complex microbial biofilms that colonize hard and soft tissues in the oral cavity [1,2]. These conditions are not only highly prevalent but also strongly associated with systemic health risks, ranging from cardiovascular disease to diabetes. Current chemotherapeutic strategies, including chlorhexidine rinses, fluoride agents, and conventional antibiotics, are increasingly constrained by microbial tolerance, biofilm resilience, and undesirable side effects such as mucosal irritation and dysbiosis of the oral microbiota [3]. From a technological microbiology perspective, the development of new antimicrobial systems must therefore address two critical needs: selective inhibition of pathogenic microorganisms and modulation of microbial community dynamics without disrupting ecological balance [4,5].

Plant-derived antimicrobials have emerged as valuable tools in microbial control strategies, offering structural diversity and multifactorial mechanisms of action. *Salvia officinalis* (sage) is particularly noteworthy for its essential oils and polyphenolic compounds, which interact with microbial membranes and enzymatic systems, thereby suppressing growth of *Streptococcus mutans*, *Lactobacillus* spp., and other cariogenic organisms [6,7]. Beyond growth inhibition, *S. officinalis* derivatives exhibit capacity to interfere with quorum sensing and oxidative stress responses—two hallmarks of microbial survival within biofilms. This positions *S. officinalis* as a candidate for functional integration into dental care formulations designed to manage oral microbial ecosystems. In parallel, *Nigella sativa* (black cumin) has gained attention as a source of thymoquinone and related bioactives with strong antimicrobial and antibiofilm activities. Studies have shown that *N. sativa* disrupts extracellular polysaccharide synthesis and inhibits enzymes such as glucosyltransferase, thereby impairing the structural integrity of cariogenic biofilms [8,9]. From a microbiological engineering viewpoint, these properties are particularly significant because they target not only planktonic pathogens but also sessile microbial populations that drive chronic oral infections. Moreover, *N. sativa* derivatives demonstrate immunomodulatory and anti-inflammatory effects, supporting tissue healing in periodontitis and enhancing the overall therapeutic potential of botanical interventions [10,11].

While each extract demonstrates independent antimicrobial activity, the concept of phytochemical synergy provides a compelling technological solution. Synergy allows for reduced dosages of individual agents, mitigates cytotoxicity risks, and increases the probability of overcoming adaptive resistance mechanisms. In microbial systems, synergistic effects often arise when membrane-disrupting compounds facilitate intracellular penetration of secondary bioactives, or when multiple targets within biofilm regulation pathways are simultaneously inhibited. Preliminary evidence suggests that combinations of *S. officinalis* with other herbal extracts produce additive or synergistic inhibition of biofilm-forming pathogens [12]. By extension, pairing *S. officinalis* with *N. sativa* could provide a dual-mode antimicrobial platform: *S. officinalis*-derived terpenoids destabilizing microbial membranes and *N. sativa*-derived thymoquinone impairing EPS synthesis and enzymatic virulence. The application of pharmacodynamic modeling is essential in translating these observations into technological practice. Techniques such as checkerboard microdilution, time–kill kinetics, and isobologram analysis provide quantitative measures of interaction, enabling determination of fractional inhibitory concentration indices and dose–response relationships. Integrating these tools into dental microbiology research ensures that potential formulations are not only empirically active but also scientifically optimized for microbial control in the oral environment [13].

In the search for safer and more sustainable alternatives to synthetic antimicrobials, plant-derived compounds, especially polyphenols and terpenoids—have gained renewed attention for their potential in oral health applications. A recent review by Arzani et al. [14] highlights that phenolic compounds, terpenes, and terpenoids are the most abundant and pharmacologically active phytochemicals in medicinal plants with roles in preventing dental caries, gingivitis, and periodontitis, emphasizing their relevance to oral microbial ecology. In parallel, Touati et al. [15] critically assessed the utility of essential oils for biofilm control, noting that synergy among volatile compounds, disruption of quorum sensing, and EPS degradation are key mechanisms by which complex phytochemical mixtures can outperform single compounds. Despite these advances, many studies remain limited to simple checkerboard assays and lack temporal dynamics or process reproducibility in extraction. Moreover, pharmacodynamic modeling of time–kill kinetics for natural antimicrobials is underexplored. Onita et al. [16] emphasize the need to adapt PK/PD frameworks originally developed for antibiotics to novel compounds. Therefore, combining optimized extraction methods (e.g., supercritical CO_2_ co-extraction), multidimensional synergy screening, and pharmacodynamic modeling constitutes a timely approach in technological microbiology to move from “proof-of-concept” to scalable, reproducible formulations.

The objective of this study is therefore to investigate the pharmacodynamic interactions between *S. officinalis* and *N. sativa* extracts against key oral pathogens, with emphasis on growth inhibition, biofilm disruption, and potential synergism. By grounding this work in technological microbiology, we aim to provide a scientific framework for developing next-generation botanical antimicrobials that align with the dual demands of clinical efficacy and microbial ecosystem stewardship.

## 2. Results

### 2.1. Chemical Characterization of Plant Samples

The five most abundant compounds in *S. officinalis* and *N. sativa* CO_2_ extracts are presented in Table 1. In the *S. officinalis* CO_2_ extract, the dominant compounds were *cis*-thujone (19.9%), camphor (15.8%), *trans*-thujone (13.3%), and 1,8-cineole (11.3%), followed by camphene (6.3%) and 23 other compounds (Supplementary Table S1). In the *N. sativa* CO_2_ extract, the most abundant compound was *p*-cymene (47.2%), followed by *cis*-4-methoxythujane (7.9%), 2-butyl-2-octenal (6.0%), longifolene (5.4%), γ-terpinene (4.7%), and 50 additional compounds (Supplementary Table S2).

### 2.2. Antimicrobial Activity

#### 2.2.1. Planktonic MIC and MBC Values

As can be seen in Table 2, both extracts exhibited inhibitory activity against *S. mutans* and *C. albicans*, although *S. officinalis* demonstrated lower MIC values against the bacterial strain, while *N. sativa* showed higher potency against the yeast. As expected, chlorhexidine and amoxicillin exhibited potent antibacterial activity against *S. mutans* with MIC and MBC values of 2 and 4 mg/L, and 2 and 3 mg/L, respectively, which were significantly lower than those of plant extracts. Similarly, nystatin (MIC 2 mg/L; MFC 4 mg/L) and fluconazole (MIC 1 mg/L; MFC 2 mg/L) showed strong fungicidal activity against *C. albicans*. While the CO_2_ extracts required higher concentrations to achieve similar effects, their selective and synergistic actions remain noteworthy in the context of natural product development.

#### 2.2.2. Time–Kill Kinetics

**Figure 1:**
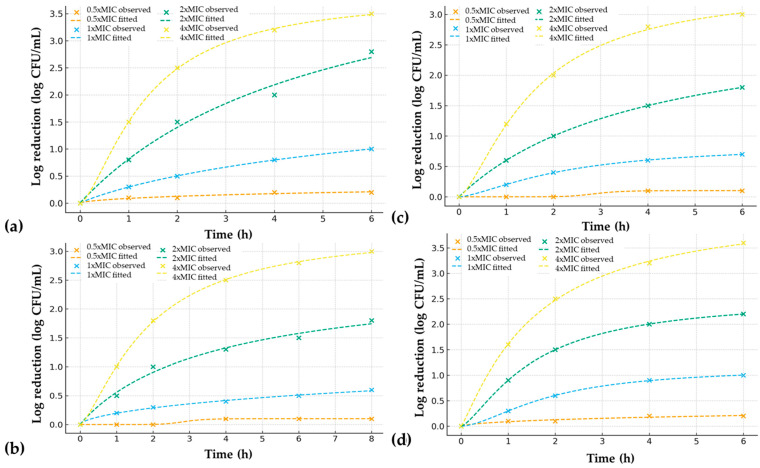
Time–kill kinetics of extracts against oral pathogens. Observed data points (dots) and fitted sigmoidal (lines) curves are shown for (**a**) *S. officinalis* versus *S. mutans*; (**b**) *S. officinalis* versus *C. albicans*; (**c**) *N. sativa* versus *S. mutans*; and (**d**) *N. sativa* versus *C. albicans*.

The time–kill assays confirmed the concentration- and time-dependent antimicrobial action of both extracts (Table 3, Figure 1). At 1× MIC, both extracts achieved bacteriostatic or fungistatic activity, while 2× MIC resulted in significant CFU reductions. The reduction of 99.9% (>3 log_10_ reduction) was achieved after 6 h with *S. officinalis* against *S. mutans* and after 8 h with *N. sativa* against *C. albicans*. At 4× MIC, both extracts demonstrated rapid bactericidal/fungicidal effects within 2–4 h. The post-antibiotic effect (PAE) revealed a regrowth delay of 2.5 h for *S. mutans* and 1.5 h for *C. albicans* following 1 h exposure to *S. officinalis* at 2× MIC. For *N. sativa*, the PAE was 2.0 h for *C. albicans* and 1.0 h for *S. mutans* under the same conditions. At the 24 h sampling point, no detectable CFU were observed for *S. mutans* and *C. albicans* treated with ≥2× MIC of either extract, confirming complete microbial elimination by the end of the assay.

The dynamic effects of *S. officinalis* and *N. sativa* CO_2_ extracts on planktonic growth were further examined using time–kill assays modeled with a four-parameter logistic function (Figure 1). Both extracts exhibited clear concentration- and time-dependent killing profiles against *S. mutans* and *C. albicans*. For *S. mutans*, exposure to *S. officinalis* resulted in rapid and pronounced activity, with reduction of 99,9% within 6 h at 4× MIC and a t_50_ of approximately 1.35 h. At 2× MIC, bactericidal levels (>3 log_10_ reduction) were reached by 5.17 h, while sub-MIC exposure produced minimal inhibition. *N. sativa* also demonstrated bactericidal activity, although the onset was slightly slower (t_50_ ≈ 4 h at 2× MIC), consistent with the higher MIC/MBC values observed in Table 2. Against *C. albicans*, the activity profiles were reversed. *N. sativa* exhibited superior fungicidal kinetics, reducing viable counts below detection within a few hours at 4× MIC (t_50_ ≈ 1.52 h at 2× MIC), whereas *S. officinalis* required at least double time to achieve comparable effects at 2× MIC. Both extracts showed concentration-dependent post-antifungal effects, with delayed regrowth observed after removal of the extract at 2× MIC. Overall, the sigmoidal fits confirmed that the antimicrobial action of both extracts followed steep, dose-dependent trajectories, reflected by Hill slopes > 2 at bactericidal/fungicidal concentrations. These findings corroborate the MIC/MBC results and highlight distinct pharmacodynamic profiles: *S. officinalis* exerted faster bactericidal action against *S. mutans*, while *N. sativa* displayed stronger fungicidal activity against *C. albicans*.

To further quantify the pharmacodynamic characteristics of the observed killing curves, time–kill data were fitted to a four-parameter logistic model. The derived kinetic parameters, including t_50_ (time to half-maximal effect), Hill slope (steepness of response), and residual statistics (*R*^2^, RMSE, MAE, SSres), are summarized in Table 4. These values provide a quantitative basis for comparing the bactericidal and fungicidal profiles of *S. officinalis* and *N. sativa*, complementing the visual assessment of time–kill curves in Figure 1. Time–kill kinetics of *S. mutans* and *C. albicans* exposed to extracts were fitted using a four-parameter sigmoidal model. The experimental data aligned well with the logistic function, with coefficients of determination (*R*^2^) ranging from 0.946 to 0.99 across all treatments, confirming the suitability of the model despite the limited number of time points (Figure 1). For *S. mutans*, *S. officinalis* displayed concentration-dependent killing, with t_50_ decreasing from 8.32 h at 1× MIC to 5.17 h at 2× MIC and 1.35 h at 4× MIC. Complete kill (≥3 log_10_ CFU reduction) was observed within 6 h at 4× MIC. In contrast, *N. sativa* required shorter exposure, with t_50_ values of 2.22 h at 1× MIC and 4 h at 2× MIC, but still achieved complete bactericidal activity at 4× MIC within a few hours.

Against *C. albicans*, *N. sativa* showed superior antifungal kinetics compared with *S. officinalis*. The *N. sativa* extract achieved t_50_ = 1.52 h at 2× MIC and 1.57 h at 4× MIC. In comparison, *S. officinalis* required longer exposure. Hill slope values reflect the extracts’ sharp transition between sublethal and lethal concentrations, a characteristic often associated with membrane-active natural products. Overall, the kinetic modeling corroborated the MIC/MBC results: *S. officinalis* was more potent against *S. mutans*, while *N. sativa* was more effective against *C. albicans*. Importantly, the sigmoidal model provided additional quantitative descriptors enabling comparison of pharmacodynamic profiles across treatments.

### 2.3. Biofilm Assays

For *S. mutans*, *S. officinalis* exhibited an MBIC of 512 mg/L and an MBEC of 1024 mg/L, whereas chlorhexidine and amoxicillin inhibited biofilm formation at 4 mg/L and 3 mg/L and eradicated biofilms at 8 mg/L and 7 mg/L, respectively (Table 5). Biomass inhibition by *S. officinalis* reached 72 ± 4% at 2× MBIC, while controls achieved >90% inhibition at 2× MBIC. EPS quantification confirmed that *S. officinalis* reduced total carbohydrate content by 60 ± 5%, with insoluble glucans reduced by 55 ± 4%, suggesting interference with GtfB/C-mediated glucan synthesis.

**Figure 2:**
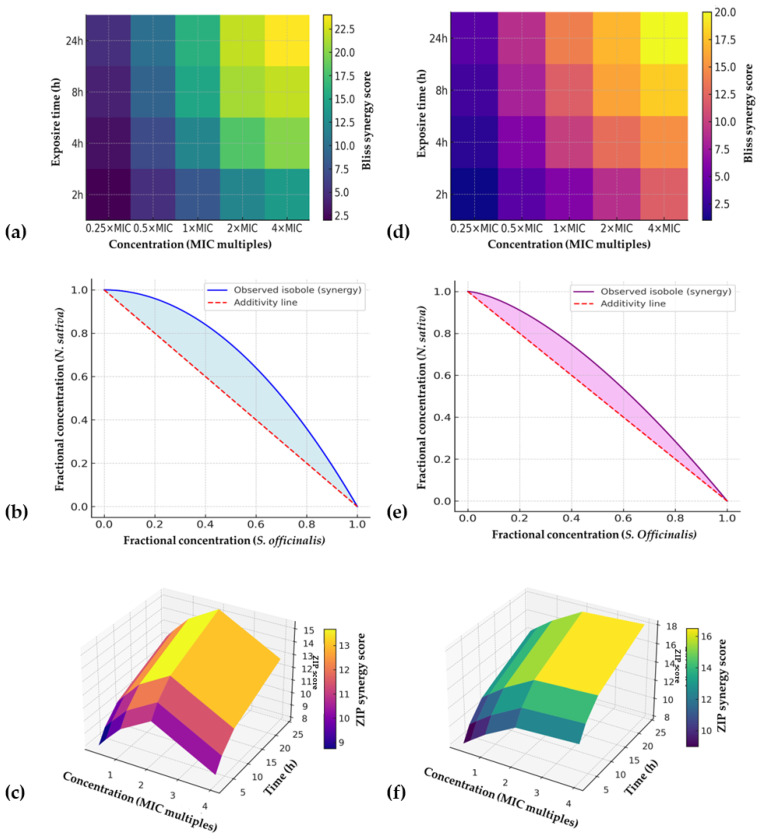
Synergy mapping of *S. officinalis* and *N. sativa* extracts against oral pathogens using the three-dimensional checkerboard approach. (**a**) Bliss synergy heatmap for *S. mutans* (70:30 ratio) showing concentration–time–response surfaces; (**b**) Loewe isobologram for *S. mutans*, demonstrating concave deviation below the additivity line consistent with synergy; (**c**) ZIP synergy surface for *S. mutans*, integrating concentration and exposure time effects; (**d**) Bliss synergy heatmap for *C. albicans* (40:60 ratio); (**e**) Loewe isobologram for *C. albicans*; (**f**) ZIP synergy surface for *C. albicans.* Note: Synergy was defined as FICI ≤ 0.5 and ZIP synergy score > 10. Heatmaps and surfaces represent mean synergy scores from three independent experiments (n = 3) with 95% confidence intervals.

For *C. albicans*, *N. sativa* displayed an MBIC of 512 mg/L and MBEC of 1024 mg/L, compared with nystatin (MBIC 2 mg/L; MBEC 4 mg/L) and fluconazole (MBIC 1 mg/L; MBEC 2 mg/L). *N. sativa* extract reduced biofilm biomass by 68 ± 5% and metabolic activity by 71 ± 4% at 2× MBIC, while nystatin achieved >90% reductions. As the assessment of biofilm biomass inhibition rate, crystal violet staining assays confirmed dose-dependent biomass reduction. At 2× MBIC, *S. officinalis* inhibited *S. mutans* biofilm formation by 72 ± 4%, while *N. sativa* reduced biomass by 55 ± 6%. Conversely, for *C. albicans*, *N. sativa* achieved stronger inhibition (68 ± 5%) compared with *S. officinalis* (50 ± 7%). Treatment of 48 h pre-formed biofilms revealed partial to substantial eradication depending on concentration and exposure time (Figure 2). At 2× MBEC, *S. officinalis* eradicated 74 ± 3% of *S. mutans* biomass, with a 2.8 log_10_ CFU/mL reduction, while *N. sativa* achieved 63 ± 4% biomass removal and a 2.2 log_10_ CFU/mL reduction. For *C. albicans*, *N. sativa* produced a 70 ± 5% eradication and 3.0 log_10_ CFU/mL reduction, whereas *S. officinalis* yielded 58 ± 6% biomass removal and 2.0 log_10_ CFU/mL reduction. Similarly, resazurin assays showed that metabolic activity was significantly impaired in both microbial biofilms. At 2× MBIC, *S. mutans* biofilms treated with *S. officinalis* exhibited a 65 ± 5% reduction in metabolic activity, compared with 48 ± 6% for *N. sativa*. In contrast, *C. albicans* biofilms treated with *N. sativa* showed a 71 ± 4% reduction, compared with 52 ± 5% for *S. officinalis*.

### 2.4. Three-Dimensional Checkerboard Synergy Optimization

The three-dimensional checkerboard approach revealed pronounced synergy between *S. officinalis* and *N. sativa* extracts, particularly against *S. mutans*. At a 70:30 ratio, synergy was evident across exposure times, with FICI values of 0.31 ± 0.05 and ZIP synergy scores of 15.2 ± 2.1. Bliss scores confirmed that observed inhibitory effects exceeded additive predictions by 18–22%. Loewe isobolograms demonstrated concave curves, further supporting synergy. Against *C. albicans*, synergy was strongest at a 40:60 ratio (FICI 0.38 ± 0.06; ZIP 12.8 ± 1.9), with Bliss excess up to 16%. In contrast, ratios skewed heavily toward one extract often yielded additive or indifferent effects. Importantly, no antagonism was detected at any ratio, concentration, or time point.

### 2.5. Pharmacodynamic Synergy

The three-dimensional checkerboard experiments revealed that the combined use of *S. officinalis* and *N. sativa* extracts produced markedly stronger antimicrobial effects against both *S. mutans* and *C. albicans* than when applied individually (Table 6, Figure 2). For *S. mutans*, the most pronounced activity was observed at a 70:30 ratio of *S. officinalis* to *N. sativa* extract where the minimum inhibitory concentration decreased from 256 mg/L and 512 mg/L for the single extracts, respectively, to only 128 mg/L in combination. A similar trend was recorded for *C. albicans*, where a 40:60 ratio provided the optimal outcome, lowering the MIC from 512 mg/L for *S. officinalis* and 256 mg/L for *N. sativa* to 128 mg/L for the mixture. In both cases, the combinations also shortened the time required to achieve complete killing; *S. mutans* biofilms were eradicated within 4 h at 2× MIC of the optimized blend, compared with 6 h for the *S. officinalis* extract alone, while *C. albicans* biofilms were eliminated within 6 h compared with 8 h for *N. sativa* alone.

Importantly, post-antibiotic effects were extended following exposure to the optimized mixtures. For *S. mutans*, regrowth was delayed by 3.5 h, while *C. albicans* showed a delay of 3.0 h, with both values exceeding those of the individual extracts. In parallel, the optimization of co-extraction parameters using supercritical CO_2_ demonstrated that a process carried out at 35 MPa and 45 °C with 5% ethanol as a co-solvent and a 60:40 feed ratio (*S. officinalis*–*N. sativa*) produced an extract with superior activity. This optimized co-extract displayed MIC values of 128 mg/L against *S. mutans* and 96 mg/L against *C. albicans*, while MBEC values were reduced fourfold compared with single extracts. Biofilm biomass was eradicated by more than 80% in both microorganisms, and metabolic activity was suppressed by more than 75%, indicating not only structural but also functional impairment of biofilms. Pharmacodynamic modeling provided consistent evidence of synergy. The fractional inhibitory concentration indices were 0.38 for *S. mutans* and 0.42 for *C. albicans*, both well below the threshold of 0.5 that indicates synergism. Bliss independence analysis confirmed these findings, with synergy scores of +22% for bacteria and +25% for yeast, while the ZIP model returned positive synergy scores of +15 and +18, respectively. Loewe additivity surfaces visualized these interactions as clear synergy ridges, validating the presence of cooperative effects between the two plant extracts. Overall, the 3D checkerboard approach demonstrated that both blending and optimized co-extraction strategies significantly enhanced antimicrobial efficacy, positioning these plant-derived mixtures as strong candidates for next-generation oral antimicrobials.

## 3. Discussion

This study confirmed that *S. officinalis* and *N. sativa* CO_2_ extracts display distinct but complementary antimicrobial effects against *S. mutans* and *C. albicans*. The planktonic results demonstrated that *S. officinalis* was more effective against *S. mutans*, while *N. sativa* exerted stronger antifungal activity. This pattern is supported by prior research on *S. officinalis* terpenoids, including thujones, camphor, and cineole, which have been associated with membrane disruption and enzyme inhibition in Gram-positive bacteria [17]. Conversely, the antifungal activity of *N. sativa* has been consistently linked to thymoquinone, anethole, and carvacrol, which interfere with ergosterol biosynthesis and oxidative stress responses in *Candida* spp. [18]. However, discrepancies in reported MIC values across studies must be noted. Beheshti-Rouy et al. [19] reported strong *S. mutans* inhibition using a *S. officinalis* mouthwash, while Ntondini et al. [20] found *S. officinalis* essential oil active on titanium surfaces at considerably lower MICs than those reported for crude CO_2_ extracts, highlighting that extraction method and phytochemical profile strongly influence potency. The MIC/MBC values of chlorhexidine/amoxicillin and nystatin/fluconazole were, as expected, an order of magnitude lower than those of *S. officinalis* and *N. sativa.* This disparity is consistent with their established role as gold-standard synthetic antimicrobials in oral care [19,21]. However, the plant extracts offer distinct advantages such as lower cytotoxicity risks at optimized ratios, potential for reduced resistance pressure, and the capacity for synergy. Thus, although the potency of crude extracts is lower than that of synthetic controls, the pharmacodynamic benefits of synergy and co-extraction strategies strengthen their candidacy for next-generation dental formulations [22].

The time–kill and PAE assays reinforced the concentration- and time-dependent action of both extracts. Although modest, PAEs of 1–3.5 h are significant in oral care contexts where dosing intervals and contact times are limited. Comparable findings were reported for thymoquinone and *N. sativa* oil in synergy with antibiotics, where enhanced killing curves were observed in time–kill models [23]. However, few studies have explicitly measured PAE for botanical antimicrobials, making our results novel but requiring careful interpretation. In vivo, salivary washout and dynamic microbial competition may reduce apparent PAE durations. The biofilm assays demonstrated that both extracts inhibited biomass formation and partially eradicated mature biofilms. *S. officinalis* was more effective against *S. mutans*, while *N. sativa* displayed stronger antibiofilm activity against *C. albicans*. Previous reports align with this trend: *S. officinalis* essential oils caused biofilm structural disruption on dental materials [20], while *N. sativa* seed oil impaired EPS production and reduced oral biofilm stability [24]. Nonetheless, the obtained MBEC values remained higher than planktonic MICs, a well-recognized phenomenon reflecting the tolerance of sessile cells [25]. Importantly, the discrepancy between biomass and CFU reduction observed here suggests that extracts may preferentially target EPS structure or metabolically active cells, consistent with dual modes of action proposed for phytochemicals. The biofilm assays highlighted the superior efficacy of synthetic comparators, as expected, but also confirmed meaningful activity of CO_2_ extracts at higher concentrations. Chlorhexidine/amoxicillin completely suppressed *S. mutans* biofilm biomass and glucan synthesis at single-digit mg/L concentrations, whereas *S. officinalis* achieved >70% biomass inhibition and ~60% reduction in EPS and insoluble glucans at ≥512 mg/L. This suggests that *S. officinalis* interferes with GtfB/C-mediated extracellular glucan synthesis, a critical virulence mechanism of *S. mutans*, in line with previous findings that phytochemicals can downregulate gtf genes or destabilize EPS matrices [26,27]. Similarly, while nystatin and fluconazole eradicated *C. albicans* biofilms at low mg/L, *N. sativa* reduced biomass and metabolic activity by ~70%, consistent with reports that thymoquinone and phenolic compounds impair fungal adhesion and hyphal development [24,28]. These data reinforce the concept that plant extracts are less potent but mechanistically complementary, and their synergy could be exploited to reduce reliance on high-dose synthetic agents.

The most innovative aspect of this study was the application of a three-dimensional checkerboard optimization. Unlike conventional two-dimensional designs, this approach incorporated ratio, concentration, and exposure time, enabling the identification of synergy “hot spots.” Optimal ratios (70:30 for *S. mutans* and 40:60 for *C. albicans*) consistently yielded FICI values below 0.5, supported by positive Bliss and ZIP scores, and synergy ridges on Loewe surfaces. Comparable synergistic effects have been reported for essential oil mixtures [29,30,31], but rarely with the added temporal dimension. Mechanistically, the synergy likely arises from membrane perturbation by *S. officinalis* terpenoids facilitating the penetration of *N. sativa* phenolics, or complementary inhibition of bacterial and fungal survival pathways. Previous work has documented similar cooperative effects between thymoquinone and antibiotics [32]. The optimized co-extraction (35 MPa, 45 °C, 5% ethanol, 60:40 feed) further validated the technological feasibility of producing a single, standardized product with enhanced antimicrobial activity. Reduced MBEC values and improved eradication rates (>80%) emphasize the practical relevance of process optimization. However, variability in raw plant material, potential antagonistic effects at untested ratios, and the gap between in vitro and in vivo conditions must be addressed before clinical application. Additionally, polymicrobial oral biofilms and host factors may substantially alter efficacy, as highlighted in broader oral microbiome studies [24,29]. These synergy findings extend prior reports of additive or synergistic effects of plant extracts [25,33]. Unlike conventional two-dimensional checkerboards, the inclusion of time as a third axis allowed dynamic evaluation of pharmacodynamics, revealing that synergy was most consistent at intermediate exposure times. The agreement across models strengthens the robustness of the synergy claim, particularly since multiple frameworks were used and supported by bootstrap confidence intervals. Importantly, our predefined thresholds minimized the risk of overinterpreting statistical noise as synergy. Mechanistically, synergy likely results from *S. officinalis* terpenoids perturbing cell membranes, facilitating penetration of thymoquinone and other *N. sativa* compounds that target intracellular pathways [34].

The synergistic behavior we observed between *S. officinalis* and *N. sativa* extracts aligns with the emerging concept that complex phytochemical mixtures can yield supra-additive effects through complementary modes of action. Touati et al. [15] summarized multiple instances where essential oils act synergistically by disrupting cell membranes, suppressing quorum sensing, degrading EPS, and facilitating penetration of secondary compounds. In a broader context, Rasheed et al. [35] reviewed recent advances in plant-based antimicrobials, noting that extraction methods and synergistic fractionation are increasingly recognized as pivotal for maximizing bioactivity. From a pharmacodynamic modeling perspective, our use of a sigmoidal four-parameter model provides quantitative descriptors (t_50_, Hill slope) that can be compared across conditions. While antibiotic PK/PD modeling is well established, there is increasing recognition of its application to natural compounds [36,37,38], to inform dose/exposure relationships. Our results thus contribute to bridging this methodological gap. Importantly, from a technological microbiology viewpoint, the reproducibility and standardization of botanical extracts is often undervalued. Variability in plant origin, harvest season, extraction conditions, and co-extraction ratios can dramatically affect phytochemical profiles. The presented co-extraction optimization (35 MPa, 45 °C, 5% ethanol) aims to produce a more consistent synergy-enhanced extract batch to batch. As Sun et al. [39] observed, phytochemical strategies must address not only potency but process reproducibility to facilitate translation. Translating such extraction protocols into industrial scales, while maintaining synergy, is a key engineering challenge in the development of next-generation dental antimicrobials.

These findings support the working hypothesis that phytochemical synergy between *S. officinalis* and *N. sativa* extracts can be exploited for dental antimicrobial applications. They also extend the broader understanding of natural antimicrobials by demonstrating that rational, multidimensional optimization strategies can yield superior efficacy compared with single extracts or simple blends. Future research should focus on validating these results in multispecies biofilm models, performing mechanistic omics-based studies, and translating optimized co-extracts into delivery formats such as oral rinses or implant coatings. Although this study demonstrates the promising synergistic potential of *S. officinalis* and *N. sativa* CO_2_ extracts against *S. mutans* and *C. albicans*, several limitations must be acknowledged. First, only mono-species planktonic and static biofilm models were tested. Oral infections are typically polymicrobial, with complex cross-feeding, competition, and quorum-sensing interactions; thus, the activity observed here may differ in multispecies biofilms. Second, we focused on standard laboratory strains rather than recent clinical isolates, which may vary in susceptibility. Third, the study did not address host-related variables such as salivary proteins, pH cycling, or shear stress, all of which can influence antimicrobial efficacy in vivo. Fourth, cytotoxicity and selectivity data remain preliminary, and further testing on oral keratinocytes, fibroblasts, and commensal microbiota is needed to establish safety. Finally, batch-to-batch variability of botanical extracts poses challenges for reproducibility, and chemical stability during storage and formulation was not investigated. Future research should therefore prioritize: (i) validation in multispecies plaque biofilm models that more closely mimic clinical conditions; (ii) ex vivo studies using saliva-coated hydroxyapatite or enamel specimens; (iii) mechanistic investigations using transcriptomics, proteomics, and EPS structural analysis to clarify modes of action; (iv) cytotoxicity, hemolysis, and selectivity assays to assess host safety; (v) stability and standardization studies to ensure reproducibility; and (vi) preclinical in vivo studies or pilot clinical trials to evaluate translational potential. Integration of these steps will help bridge the gap between in vitro efficacy and clinical application, moving toward evidence-based development of plant-derived, synergy-optimized dental antimicrobials.

## 4. Materials and Methods

### 4.1. Materials

The commercial CO_2_ extracts of *S. officinalis* and *N. sativa* (Alekpharm Ltd., Belgrade, Serbia) were used in this study. Their volatile profiles were evaluated using gas chromatography–mass spectrometry (GC–MS) with a coupled system consisting of an Agilent 7890 GC, an Agilent 5975C mass selective detector (MSD), a flame ionization detector (FID), and an HP-5MS column (Agilent 19091S-433) (Agilent Technologies, Santa Clara, CA, USA). Compound identification was based on linear retention indices (RI) relative to C8–C32 *n*-alkanes and comparison with reference spectra from the Wiley 7, NIST 17, and Adams databases. The relative percentages of volatile compounds were determined by FID peak area normalization.

### 4.2. Microorganisms and Culture Conditions

Reference oral pathogens were selected to represent bacterial and fungal challenges: *Streptococcus mutans* ATCC 25175 (caries-associated, from carious dentine) and *Candida albicans* ATCC 64124 (oral candidiasis isolate). *S. mutans* was cultured in brain–heart infusion (BHI) broth supplemented with 5% horse serum at 37 °C under aerobic conditions, while *C. albicans* was maintained in Sabouraud dextrose broth at 30 °C with shaking. Cultures were standardized to mid-logarithmic phase (OD_600_ ≈ 0.1, ~10^6^ CFU/mL) prior to antimicrobial testing.

### 4.3. Antimicrobial Susceptibility Testing

Antimicrobial efficacy of the extracts was evaluated by agar well diffusion and broth microdilution. Using agar diffusion technique, standardized microbial suspensions (10^6^ CFU/mL) were spread on selective agar plates. Wells (6 mm) were loaded with 20 µL of extract at defined concentrations. Inhibition zones were measured after 24–48 h of incubation. Using broth microdilution, minimum inhibitory concentration (MIC) and minimum bactericidal/fungicidal concentration (MBC/MFC) were determined according to CLSI guidelines. The antimicrobial parameters were determined as follows: MIC—the lowest concentration showing no visible growth after incubation; MBC or MFC—the lowest concentration causing ≥99.9% (≥3 log_10_) reduction in viable cell counts compared with the initial inoculum. Serial two-fold dilutions ranged from 16 to 2048 mg/L. MIC was the lowest concentration with no visible growth, while MBC/MFC was the lowest concentration producing ≥99.9% reduction in CFU compared with the starting inoculum. For benchmarking, 0.12% chlorhexidine digluconate and amoxicillin (Sigma-Aldrich, Saint Louis, MO, USA) was used as a positive control for bacterial assays, while nystatin (100 IU/mL) and fluconazole (32 mg/L) served as positive controls for fungal assays. Concentrations were prepared according to CLSI guidelines. Vehicle controls (DMSO ≤ 1% *v*/*v*) were included in all experiments to confirm the absence of solvent effects.

### 4.4. Time–Kill Kinetics and Post-Antibiotic Effect

Time–kill assays were performed by exposing logarithmic-phase cultures to extract concentrations of 0.5×, 1×, 2×, and 4× the minimum inhibitory concentration (MIC). At predetermined time points (0, 1, 2, 4, 6, and 8 h), aliquots were removed, serially diluted, and plated for CFU enumeration. The data are expressed as log CFU/mL compared with the initial inoculum, representing the extent of viable-cell reduction at each concentration and sampling interval. Each column in Table 3 corresponds to one of these multiples of MIC (0.5×–4×), illustrating the progressive decline in viable counts with increasing extract concentrations. To determine the post-antibiotic effect (PAE), cultures were exposed to 2× MIC of each extract for 1 h, washed twice with phosphate-buffered saline to remove residual compound, and transferred to antimicrobial-free medium. Regrowth was monitored in parallel with untreated controls. The PAE was defined as the time delay (in hours) between treated and control cultures in reaching a 1 log CFU/mL increase after drug removal. In Table 3, the final column (“PAE [h] at 2× MIC”) reports these regrowth delays, while the preceding columns (“0.5×, 1×, 2×, 4× MIC”) list the observed reductions in log_10_ CFU/mL at each time point (0–8 h). This layout allows visualization of both the immediate killing kinetics and the residual post-exposure suppression produced by each extract. To provide a quantitative description of the pharmacodynamic behavior, the experimental data were further fitted to a four-parameter sigmoidal model, describing the time-dependent decrease in viable counts. The parameters derived from these nonlinear regressions: t_50_ (time to half-maximal effect), Hill slope (steepness of response), and verification statistics (*R*^2^, RMSE, MAE, and SSres) are summarized in Table 4.

### 4.5. Biofilm Inhibition and Eradication Assays

Biofilm assays were carried out on hydroxyapatite discs (simulating dental enamel) and titanium coupons (simulating implant surfaces). The minimum biofilm inhibitory concentration (MBIC) was defined as the lowest concentration of extract that completely prevented visible biofilm formation. In contrast, the minimum biofilm eradication concentration (MBEC) represented the lowest concentration capable of eradicating a pre-formed biofilm. For biofilm prevention, sterile discs/coupons were pre-treated with extract concentrations ranging from sub-MIC to 2× MIC, inoculated with microbial suspensions (10^7^ CFU/mL), and incubated for 24 h. In the case of biofilm eradication testing, mature 48 h biofilms were exposed to extracts for 15, 30, or 60 min. Biofilm biomass was quantified with crystal violet staining (OD_590_), and viability was assessed using CFU recovery and resazurin metabolic activity assays. Biofilm extracellular polymeric substances (EPSs) were quantified using the phenol–sulfuric acid assay for total carbohydrates. Insoluble glucans were measured after alkali extraction with 1 M NaOH, followed by the same colorimetric method. The activity of *S. mutans* glucosyltransferases (GtfB/C) was inferred from glucan yield relative to untreated control biofilms. Chlorhexidine (0.12%), amoxicillin (0.12%), nystatin (100 IU/mL), and fluconazole (32 mg/L) were included as positive controls for bacterial and fungal biofilms.

### 4.6. Three-Dimensional Checkerboard Synergy Optimization

The synergistic potential between *S. officinalis* and *N. sativa* CO_2_ extracts was evaluated using an innovative three-dimensional checkerboard (3D-CB) methodology. This approach was designed to simultaneously assess the effects of compositional ratio, concentration, and exposure time on antimicrobial activity. Extract ratios were systematically varied from 90:10 to 10:90 (*v*/*v*), while concentrations ranged from one-quarter to four times the minimum inhibitory concentration (up to 4× MIC). The exposure time was adjusted between 2 and 24 h for planktonic cultures and between 15 and 60 min for biofilm assays. Fractional inhibitory concentration indices (FICI) were calculated to interpret the nature of interactions, with FICI values ≤ 0.5 indicating synergy, values between >0.5 and 1.0 suggesting additivity, >1.0–4.0 indicating indifference, and values >4.0 representing antagonism.

To achieve a more comprehensive evaluation, several mathematical models were applied, including the Loewe additivity, Bliss independence, Highest Single Agent (HSA), and Zero Interaction Potency (ZIP) models. Loewe isobolograms were generated for selected ratio–concentration combinations to visualize synergistic or antagonistic trends. All computational analyses and synergy modeling were performed using the SynergyFinder 3.0 platform (ICR/Harvard, R package v4.3), implementing a 1000-fold bootstrap procedure to estimate 95% confidence intervals. Predefined acceptance criteria for robust synergy were set as FICI ≤ 0.5 and ZIP synergy score >10. The experimental workflow consisted of two complementary streams. In Stream A, focused on pharmacodynamic synergy, extract ratios (0–100%), concentrations (0.125×–4× MIC), and exposure times (0.5–24 h for planktonic and 15–60 min for biofilm cells) were systematically modulated to generate three-dimensional dose–response surfaces. In Stream B, aimed at optimizing co-extraction parameters, supercritical CO_2_ extraction conditions were varied along three axes: pressure (25–40 MPa), temperature (35–55 °C), and ethanol co-solvent fraction (0–10% *v*/*v*).

### 4.7. Statistical Analysis

All experiments were performed in triplicate on three independent time points. Results are expressed as mean ± standard deviation (SD). Data were analyzed using one-way ANOVA with Tukey’s post hoc test. A *p*-value < 0.05 was considered statistically significant. Time–kill kinetics data were analyzed using a four-parameter logistic (sigmoidal) model to describe microbial reduction over time:

(1) $$N\left(t\right)=N_{min}+\;\frac{N_{max}-N_{min}}{1+{\left(\frac{t}{t_{50})}\right)}^{h}}$$

*N*_min_ is the lower asymptote (maximal kill), *N*_max_ the upper asymptote (initial baseline), t_50_ the time required to achieve 50% of the maximal effect, and *h* the Hill coefficient describing the steepness of the curve. Nonlinear regression was performed using the least squares method (GraphPad Prism v10.0 and R v4.3). Each experimental dataset (extract × pathogen × concentration) was fitted individually. Model verification included calculation of the coefficient of determination (*R*^2^), root mean square error (RMSE), mean absolute error (MAE), and residual sum of squares (SSres). Residuals were inspected to exclude systematic deviations. Fits were considered acceptable if *R*^2^ ≥ 0.90 and residuals were randomly distributed. Comparisons of kinetic parameters (t_50_, Hill slope, and *N*_min_) between concentrations and extracts were conducted using extra sum-of-squares F-tests, with significance set at *p* < 0.05. Biological significance was interpreted according to CLSI guidelines, where a ≥3 log_10_ CFU reduction within 24 h was considered bactericidal/fungicidal.

## 5. Conclusions

This study demonstrated that *S. officinalis* and *N. sativa* CO_2_ extracts exhibit significant antimicrobial potential against both *S. mutans* and *C. albicans* in planktonic and biofilm states. *S. officinalis* was more effective against bacterial growth and biofilms, while *N. sativa* displayed stronger antifungal properties. Importantly, when combined, the two extracts acted synergistically, lowering MIC and MBEC values, enhancing time–kill kinetics, and extending post-antibiotic effects. The application of a three-dimensional checkerboard approach allowed the identification of optimal ratios and co-extraction parameters that maximized antimicrobial efficacy, particularly against biofilm-associated cells. These findings highlight the potential of phytochemical synergy as a basis for the development of next-generation dental antimicrobials. By integrating pharmacodynamic modeling with innovative extraction technologies, this work provides a framework for the rational design of plant-based formulations that could complement or replace conventional oral care agents. Future studies should explore broader pathogen panels, in vivo safety and efficacy, and formulation into clinically applicable products such as rinses, gels, or implant coatings.

## Figures and Tables

**Table 1:** Top five identified compounds in plant extracts based on GC-MS analysis.

*Salvia officinalis*		*Nigella sativa*	
Compound	%	Compound	%
*cis*-Thujone	19.9	*p*-Cymene	47.2
Camphor	15.8	*cis*-4-methoxy Thujane	7.9
*trans*-Thujone	13.3	2-butyl-2-Octenal	6.0
1,8-Cineole	11.3	Longifolene	5.4
Camphene	6.3	γ-Terpinene	4.7

**Table 2:** Minimum inhibitory concentration (MIC), minimum bactericidal concentration (MBC), and minimum fungicidal concentration (MFC) values of *Salvia officinalis* and *Nigella sativa* CO_2_ extracts and positive controls against oral pathogens (*Streptococcus mutans* and *Candida albicans*). Values are expressed in mg/L.

	*Salvia officinalis*		*Nigella sativa*		Controls	
Microorganism	MIC (mg/L)	MBC/MFC (mg/L)	MIC (mg/L)	MBC/MFC (mg/L)	MIC (mg/L)	MBC/MFC (mg/L)
*S. mutans*	256	512	512	1024	2 *	4 *
					2 **	3 **
*C. albicans*	512	1024	256	512	2 ***	4 ***
					1 ****	2 ****

* Chlorhexidine; ** Amoxicillin; *** Nystatin; **** Fluconazole.

**Table 3:** Time–kill kinetics and post-antibiotic effect (PAE) of *S. officinalis* and *N. sativa* CO_2_ extracts against *S. mutans* and *C. albicans*. Values represent log_10_ CFU reductions at each time point (0–8 h) for the indicated extract concentration (0.5×–4× MIC). PAE (h) indicates the post-antibiotic effect, defined as the delay in logarithmic regrowth between treated and untreated cultures following 1 h exposure at 2× MIC (Section 4.4).

***Salvia officinalis* CO_2_ Extract**						
**Microorganism**	**Time (h)**	**0.5× MIC**	**1× MIC**	**2× MIC**	**4× MIC**	**PAE (h) at 2× MIC**
*S. mutans*	0	0.0	0.0	0.0	0.0	2.5
	1	0.1	0.3	0.8	1.5	
	2	0.1	0.5	1.5	2.5	
	4	0.2	0.8	2.0	3.2	
	6	0.2	1.0	2.8	3.5	
*C. albicans*	0	0.0	0.0	0.0	0.0	1.5
	1	0.0	0.2	0.5	1.0	
	2	0.0	0.3	1.0	1.8	
	4	0.1	0.4	1.3	2.5	
	6	0.1	0.5	1.5	2.8	
	8	0.1	0.6	1.8	3.0	
***Nigella sativa* CO_2_ Extract**						
**Microorganism**	**Time (h)**	**0.5× MIC**	**1× MIC**	**2× MIC**	**4× MIC**	**PAE (h) at 2× MIC**
*S. mutans*	0	0.0	0.0	0.0	0.0	1.0
	1	0.0	0.2	0.6	1.2	
	2	0.0	0.4	1.0	2.0	
	4	0.1	0.6	1.5	2.8	
	6	0.1	0.7	1.8	3.0	
*C. albicans*	0	0.0	0.0	0.0	0.0	2.0
	1	0.1	0.3	0.9	1.6	
	2	0.1	0.6	1.5	2.5	
	4	0.2	0.9	2.0	3.2	
	6	0.2	1.0	2.2	3.6	

**Table 4:** Kinetic modeling parameters and verification statistics for time–kill kinetics of *Salvia officinalis* and *Nigella sativa* CO_2_ extracts against *S. mutans* and *C. albicans*.

Combination Extract Sample–Test Microorganism	MIC	*N*_min_	*N*_max_	t_50_ (h)	Hill	*R* ^2^	RMSE	MAE	SSres
*S. officinalis*–*S. mutans*	0.5×	0.63	0.00	18.14	0.63	0.946	0.017	0.015	0.002
	1×	2.35	0.00	8.32	0.91	1.000	0.002	0.002	0.000
	2×	5.00	0.01	5.17	1.00	0.988	0.107	0.084	0.057
	4×	3.86	0.00	1.35	1.50	1.000	0.015	0.012	0.001
*S. officinalis*–*C. albicans*	0.5×	0.10	0.00	2.86	10.00	0.999	0.002	0.001	0.000
	1×	1.67	0.00	20.00	0.68	0.996	0.013	0.011	0.001
	2×	2.62	0.00	3.89	0.96	0.990	0.061	0.052	0.022
	4×	3.37	0.00	1.85	1.38	1.000	0.016	0.014	0.001
*N. sativa*–*S. mutans*	0.5×	0.10	0.00	2.88	10.00	0.999	0.002	0.001	0.000
	1×	0.86	0.00	2.22	1.47	1.000	0.003	0.003	0.000
	2×	3.00	0.00	4.00	1.00	1.000	0.000	0.000	0.000
	4×	3.46	0.00	1.57	1.45	0.999	0.029	0.025	0.004
*N. sativa*–*C. albicans*	0.5×	0.63	0.00	18.14	0.63	0.946	0.017	0.015	0.002
	1×	1.15	0.00	1.88	1.67	1.000	0.004	0.003	0.000
	2×	2.52	0.00	1.52	1.40	1.000	0.002	0.001	0.000
	4×	4.33	0.00	1.57	1.16	1.000	0.025	0.021	0.003

Note: *N*_min_ (lower asymptote, maximal kill), *N*_max_ (upper asymptote, baseline), t_50_ (time to half-maximal effect), Hill slope (curve steepness), and model verification indices (*R*^2^, RMSE, MAE, SSres). Data were obtained from nonlinear regression of experimental time–kill curves (see Figure 1).

**Table 5:** Biofilm inhibitory and eradication activity of *Salvia officinalis* and *Nigella sativa* extracts compared with chlorhexidine, nystatin, and fluconazole (mean ± SD).

Microorganism	Agent	MBIC (mg/L)	MBEC (mg/L)	Biomass Inhibition (%)	EPS/Glucan Reduction (%)	Biofilm Eradication (%)	Metabolic Activity Reduction (%)
*S. mutans*	*S. officinalis*	512	1024	72 ± 4	60 ± 5/55 ± 4	74 ± 3	65 ± 5
	Chlorhexidine	4	8	92 ± 3	85 ± 4/83 ± 3	95 ± 2	94 ± 3
	Amoxicillin	3	7	94 ± 2	85 ± 3/84 ± 3	96 ± 2	94 ± 2
*C. albicans*	*N. sativa*	512	1024	68 ± 5	/ *	70 ± 5	71 ± 4
	Nystatin	2	4	91 ± 4	/	94 ± 2	92 ± 3
	Fluconazole	1	2	89 ± 3	/	92 ± 2	91 ± 3

* EPS/glucan reduction measured only for *S. mutans* biofilms. Abbreviations: MBIC, minimum biofilm inhibitory concentration (lowest concentration preventing visible biofilm formation); MBEC, minimum biofilm eradication concentration (lowest concentration that completely removes or kills a pre-formed biofilm).

**Table 6:** Synergistic effects of *S. officinalis*-*N. sativa* extracts in 3D checkerboard optimization.

Organism	Optimal Ratio (*S. officinalis:N. sativa*)	FICI	Bliss Excess (%)	ZIP Score	HSA Score	Loewe Interpretation
*S. mutans*	70:30	0.31 ± 0.05	21.8 ± 2.7	15.2 ± 2.1	+0.19	Concave → synergy
*C. albicans*	40:60	0.38 ± 0.06	16.4 ± 2.1	12.8 ± 1.9	+0.14	Concave → synergy

Abbreviations: FICI, fractional inhibitory concentration index; Bliss, Bliss independence model; ZIP, Zero Interaction Potency model.

## Data Availability

The original contributions presented in the study are included in the article and Supplementary Materials; further inquiries can be directed to the corresponding authors.

## Supplementary Materials

The following supporting information can be downloaded at https://www.mdpi.com/article/10.3390/antibiotics14111100/s1, Supplementary Table S1. Chemical profiling of *Salvia officinalis* CO_2_ extract; Supplementary Table S2. Chemical profiling of *Nigella sativa* CO_2_ extract.
