# Supplementary material for: Targeting Oral Pathogens with Salvia officinalis and Nigella sativa Supercritical CO2 Extracts: A Pharmacodynamic Approach and Three-Dimensional Checkerboard Synergy for Novel Dental Antimicrobials
Source: Antibiotics (Basel). 2025 Nov 2;14(11):1100. doi: 10.3390/antibiotics14111100 (PMC12649734; doi:10.3390/antibiotics14111100)
Supplement: Supplementary file 1 [file antibiotics-14-01100-s001.zip › Supplementary Table S1. Chemical profiling of Salvia officinalis CO2 extract.pdf]

Supplementary Table S1. Chemical profiling of *Salvia officinalis* CO_2 extract

Peak	Compound	RI	%
1	cis-Salvene	846	0.3
2	Tricyclene	921	0.2
3	α-Thujene	924	0.2
4	α-Pinene	931	4.3
5	Camphene	945	6.3
6	Sabinene	970	0.1
7	β-Pinene	973	3.9
8	Myrcene	987	1.0
9	α-Phellandrene	1002	0.1
10	α-Terpinene	1014	0.2
11	p-Cymene	1021	0.4
12	Limonene	1025	3.1
13	1,8-Cineole	1027	11.3
14	γ-Terpinene	1054	0.4
15	Terpinolene	1085	0.3
16	Linalool	1097	0.5
17	cis-Thujone	1103	19.9
18	trans-Thujone	1113	13.3
19	Camphor	1139	15.8
20	Borneol	1159	3.6
21	Bornyl acetate	1279	1.1
22	α-Copaene	1369	0.1
23	trans-Caryophyllene	1412	4.2
24	α-Humulene	1447	4.0
25	allo-Aromadendrene	1459	0.1
26	δ-Cadinene	1517	0.1
27	Humulene epoxide II	1601	0.4
28	13-epi-Manool	2054	0.3
