# Supplementary material for: Targeting Oral Pathogens with Salvia officinalis and Nigella sativa Supercritical CO2 Extracts: A Pharmacodynamic Approach and Three-Dimensional Checkerboard Synergy for Novel Dental Antimicrobials
Source: Antibiotics (Basel). 2025 Nov 2;14(11):1100. doi: 10.3390/antibiotics14111100 (PMC12649734; doi:10.3390/antibiotics14111100)
Supplement: Supplementary file 1 [file antibiotics-14-01100-s001.zip › Supplementary Table S2. Chemical profiling of Nigella sativa CO2 extract.pdf]

Supplementary Table S2. Chemical profiling of *Nigella sativa* CO_2 extract

Peak	Compound	RI	%
1	α-Thujene	925	1.0
2	β-Pinene	976	1.1
3	2-Pentyl furan	993	1.7
4	α-Terpinene	1017	0.1
5	p-Cymene	1025	47.2
6	γ-Terpinene	1058	4.7
7	trans-4-methoxy thujane	1098	1.2
8	Nonanal	1104	0.6
9	cis-4-methoxy thujane	1119	7.9
10	cis-Chrysanthenol	1165	0.1
11	Terpinen-4-ol	1176	0.3
12	Thymoquinone	1249	1.0
13	2E-Decenal	1262	0.4
14	iso-3-Thujanol acetate	1265	0.5
15	Bornyl acetate	1286	0.6
16	Anethole	1289	0.3
17	2E,4Z-Decadienal	1295	0.4
18	Carvacrol	1303	1.4
19	2E,4E-Decadienal	1317	1.0
20	α-Longipinene	1351	1.0
21	2E-Undecenal	1364	0.7
22	2-butyl-2-Octenal	1375	6.0
23	Hexyl hexanoate	1387	0.3
24	β-Elemene	1393	0.1
25	Tetradecane	1400	0.6
26	Longifolene	1406	5.4
27	trans-Caryophyllene	1421	0.3
28	Geranyl acetone	1455	0.1
29	ar-Curcumene	1486	0.3
30	β-Selinene	1489	0.1
31	2-Tridecanone	1498	0.1
32	Pentadecane	1502	0.1
33	β-Bisabolene	1511	0.3
34	Zonarene	1529	0.1
35	Citronellyl butanoate	1530	0.1
36	Caryophyllene oxide	1585	0.4
37	cis-7-Tetradecenal	1594	0.3
38	Hexadecane	1600	0.1
39	Tetradecanal	1613	0.2
40	ar-Turmerone	1669	0.2
41	Longiborneol acetate	1684	0.3
42	Heptadecane	1701	0.2
43	2-Pentadecanone, 6,10,14-trimethyl	1847	0.2
44	epi-Laurenene	1902	0.2
45	Methyl palmitate	1929	0.1
46	ent-Rosa-5,15-diene	1936	0.1
47	Pimaradiene	1948	1.0
48	Butyric acid, tetradecyl ester	1989	0.2
49	Kaur-15-ene	1995	0.1
50	Methyl linoleate	2102	0.7
51	Methyl oleate	2108	0.8
52	Ethyl linoleate	2173	0.1

53	1-Methylbutyl hexadecanoate	2294	0.1
54	Pentyl 9,12-octadecadienoate	2462	0.1
55	Pentyl oleate	2467	0.1
